# Exonic Variation and Its Clinical Impact in 7221 Old Order Amish

**DOI:** 10.1002/ajmg.a.64212

**Published:** 2025-08-06

**Authors:** Braxton D. Mitchell, Ebuka Onyenobi, Joshua P. Lewis, Brady Gaynor, James A. Perry, Kristin Maloney, Jeffrey R. O'Connell, Jessica Tiner, Amber L. Beitelshees, Cristopher V. Van Hout, Patrick F. McArdle, Huichun Xu, Erik G. Puffenberger, Karlla W. Brigatti, Melanie Daue, Hilary B. Whitlatch, Anna Alkelai, Alejandro A. Schäffer, John Overton, Elizabeth A. Streeten, Toni I. Pollin, Alan R. Shuldiner

**Affiliations:** 1 Department of Medicine, University of Maryland School of Medicine, Baltimore, Maryland, USA; 2 Department of Epidemiology & Public Health, University of Maryland School of Medicine, Baltimore, Maryland, USA; 3 Baltimore Veterans Administration Medical Center Geriatrics Research and Education Clinical Center, Baltimore, Maryland, USA; 4 Laboratorio Internacional de Investigatión Sobre el Genoma Humano, Universidad Nacional Autónoma de México Campus Juriquilla, Querétaro, Mexico; 5 Clinic for Special Children, Gordonville, Pennsylvania, USA; 6 Regeneron Genetics Center LLC, Tarrytown, New York, USA; 7 Cancer Data Science Laboratory, National Cancer Institute, National Institutes of Health, Bethesda, Maryland, USA

**Keywords:** Amish, exomes, founder population, pathogenic variants, population genetics

## Abstract

The Amish of Lancaster County, PA has been the focus of genetic studies for many years due to its demographic history and unique genetic makeup that includes a historical bottleneck event and subsequent genetic drift, resulting in a marked decrease in genetic diversity and increased frequency of some variants that have substantially shaped the health of the community. To characterize the coding variation in the Amish genome, we sequenced the exomes of 7221 adult community members, and in this report, we contrast genetic diversity between the Amish and Europeans from the UK Biobank. Exome sequences of 7221 Amish contained only 14% as many variants as the same number of UKB participants. This reduced genetic diversity has substantial clinical implications. We identified pathogenic (P) and likely pathogenic (LP) variants from ClinVar and a population-specific genetic screening panel and found that most of the variants present in the Amish were highly enriched, resulting in 5.2% of Amish individuals being homozygous for a recessive P/LP variant and 25.6% being heterozygous for at least one dominant P/LP variant. In 43.6% of the 2141 Amish spouse-pairs in our sample, at least one spouse was heterozygous for a P/LP dominant variant, and 24.3% of couples were autosomal recessive disease carrier couples, meaning that each of their children was at ~25% risk of inheriting two copies of that variant. Gene discovery efforts in other founder communities will likely uncover distinct P (and beneficial) variants impacting the health of these communities, with implications for all of human health.

## 1 ∣ Introduction

The Old Order Amish (OOA) of Lancaster County, PA, are a relatively recent founder population who emigrated from Central Europe, primarily present-day Switzerland, in the early 1700s. The Lancaster settlement was founded by ~400 settlers (Lee et al. 2010), and the community has now expanded its geographic footprint to multiple counties adjacent to Lancaster and grown to approximately 43,000 individuals, making it the largest Amish settlement in the United States (Young Center for Anabaptist and Pietist Studies 2025). Amish comprise one of three main sets of North American religious groups, along with Mennonites and Hutterites, classified as Anabaptist, meaning that baptism is performed in adulthood rather than in infancy. As with many other Anabaptist communities, the Amish have remained largely closed, with marriages restricted to individuals within the community. Because some early settlers had more descendants than others, some founders have a higher representation of their genomes in the present-day population. In fact, a previous coalescence analysis indicated that only 128 founders account for over 95% of the average extant genome (Lee et al. 2010).

Founder populations like the OOA have been the focus of genetic studies for many years because of their unique genetic architecture (Peltonen et al. 2000; Southam et al. 2017). Early studies in the Amish focused on mapping and characterizing rare monogenic Mendelian disorders that are enriched in this population (Francomano et al. 2003; McKusick 1978; McKusick et al. 1964). Later investigations utilizing large community study designs have also uncovered genetic variants that influence more common disorders and traits such as cardiovascular disease, lipid disorders, diabetes, and pharmacogenetics (Albert et al. 2014; Horenstein et al. 2013; Montasser et al. 2021; Pollin et al. 2008; Shen et al. 2010; Shuldiner et al. 2009). The genomic landscape of the Amish, including enrichment of high-penetrance large-effect variants, is a consequence of a bottleneck effect with a small number of founders, a lack of new genetic influx, genetic drift, and endogamy. Identification of these variants has informed human biology and therapeutic targets more generally (Pollin et al. 2008).

The limited genetic diversity of Anabaptist populations compared to European reference populations has been previously documented empirically through a whole-genome sequencing study (Hou et al. 2017). However, this prior study included only 265 individuals, among whom were Mennonites and Midwestern Amish as well as Lancaster Amish. The goals of this report are twofold. First, we comprehensively characterize the scope of variation across the exome in 7221 Amish from Lancaster County, PA by comparing the numbers and types of variants in Amish with those of 7221 individuals of European ancestry sampled from the UK Biobank (UKB). Second, we describe the clinical impact of limited genetic diversity and drift in the Amish by comparing frequencies of pathogenic (P) and likely pathogenic (LP) variants that have been associated with adverse clinical outcomes between the Amish and UKB. Third, we enumerate the proportion of Amish couples at risk for transmitting known Mendelian disorders to their offspring. Fourth, we calculate the proportion of individuals carrying P variants in American College of Medical Genetics (ACMG) clinically actionable genes. Finally, we describe nine highly enriched genetic variants identified through genome-wide association analysis in our Amish cohort, some of which have overall beneficial effects on cardiovascular health.

## 2 ∣ Methods

### 2.1 ∣ Editorial Policies and Ethical Considerations

Ethical approval for all Amish studies was obtained from the Institutional Review Board at the University of Maryland, Baltimore. Amish study participants provided written informed consent for genetic analyses of their DNA.

### 2.2 ∣ Study Participants

Amish subjects in this study were recruited through multiple protocols carried out between 1995 and 2019 as part of the University of Maryland Amish Research Program (He et al. 2020; Hsueh et al. 2000; Mitchell et al. 2008; Streeten et al. 2005). Studies were designed to assess determinants of health and disease in the community, and enrollment was open to adult volunteers throughout the Lancaster Amish community. Recruitment, carried out at the University of Maryland Amish Research Clinic in Lancaster, PA, varied from smaller studies centering on particular conditions (e.g., diabetes, longevity, bone health, and cardiovascular health) for which recruitment was focused around affected and unaffected individuals, their family members, and other community members, to larger studies that were phenotype agnostic; that is, no focused recruitment for particular diseases or health conditions (e.g., The Amish Wellness Study) (see Table S1). This report is based on 7221 Amish individuals from the community who were 18 years of age or older at the time of their recruitment across these studies and in whom exome sequencing (ES) was performed at the Regeneron Genetics Center (RGC). Based on the 2024 census of 43,640 Amish in Lancaster County (Young Center for Anabaptist and Pietist Studies 2025) and our estimate that ~50% of these individuals are aged 18 years and older, we estimate that this sample represents approximately 33%–40% of the Lancaster Amish population age 18 years and older.

For comparison with the Amish, we selected at random 7221 out of 41,245 subjects from the UKB's first 50,000 subjects with exome sequence data and who were also included in the UKB's Data-Field 22,006, and from which we selected subjects who self-identified as “White British.” From here onwards, we refer to the UK-white-British exomes as “UKB European”

### 2.3 ∣ ES and Genome Informatics

Exome capture and sequencing were performed on 7319 OOA samples at the RGC. Exome capture was performed using a slightly modified version of the xGen capture reagent available from Integrated DNA Technologies (IDT) with some additional probes. The captured libraries were sequenced on the Illumina HiSeq 2500 and NovaSeq 6000 platforms. A cloud-based pipeline that uses standard bioinformatics tools was used for sample-level data production and analysis. Raw sequence data was uploaded to DNAnexus for automated production, including sample de-multiplexing using Illumina's CASAVA software (Illumina Inc., San Diego, CA, USA). Sequence reads were mapped and aligned to the GRCh38/hg38 human genome reference assembly using BWA-mem (Li and Durbin 2009). SNP and INDEL variants were called using the weCall variant caller from GENOMICS plc. Captured fragments were sequenced to achieve a minimum of 85% of the target bases covered at 20× or greater; SNPs with call rate < 90%, and monomorphic SNPs were excluded. SNPs on X and Y chromosomes and the mitochondrial genome were also excluded. Samples failing QC metrics for contamination (*N* = 9), low coverage (*N* = 16), high levels of Mendelian inconsistencies (*N* = 27), identical or MZ twins (one of each pair, *N* = 26), gender mismatch (*N* = 13), and duplicates (*N* = 7) were excluded, leaving 7221 samples for the analysis.

ES in UKB samples was also performed at the RGC using a similar pipeline as previously described (Van Hout et al. 2020). Quality control procedures were as described above. We compared the total number of sequenced variants present between the two populations, as well as the number of variants in target regions. We defined targeted regions as exonic or splicing regions using the ANNOVAR tool (Wang et al. 2010). The exonic regions include all coding regions of the gene, while the splicing regions encompass the exon–intron boundaries important for mRNA splicing. These regions were selected for variant annotation to assess the potential functional impact on gene expression and protein function. To refine the identification of potential splice site disruptions, we further utilized SpliceAI, a deep learning-based tool, to predict the impact of variants on splice site activity (Jaganathan et al. 2019). SpliceAI provides high-confidence predictions of splice site disruptions, helping to identify variants that may alter splicing at exon–intron junctions.

### 2.4 ∣ Functional Annotation of Variants

Variants were annotated according to type (SNV, indel, or multiallelic) and predicted function. Predicted effects were inferred using ANNOVAR. Variants were designated as predicted loss-of-function (pLOF) if they gained a frameshift, premature stop codon, loss-of-stop codon, or splice site disruption. In-frame indels and missense variants were classified as non-synonymous variants.

### 2.5 ∣ Comparisons of Exonic Variants and pLOF Variants Between Amish and UKB Europeans

We compared the number and types of variants between Amish and UKB Europeans using chi-square tests and contrasted the mean number of pLOF variants per individual using the *t*-test. To illustrate the impact of genetic architecture on gene burden, we further compared the number of Amish and UKB European participants who were homozygous for pLOF variants.

### 2.6 ∣ Relatedness Among Amish and UKB European Participants

To compare the relationship types within Amish and UKB European pairs, we used the plink software package (Purcell et al. 2007) to estimate the mean identity-by-descent among pairs. To obtain finer-scale estimations of genetic relatedness within the Amish, we utilized the genetic data from ES to estimate genetic similarities within Amish pairs (Yang et al. 2011) since plink underestimates identity-by-descent relatedness for complex pedigree structures.

### 2.7 ∣ Clinical Impact of Limited Genetic Diversity and Drift in the Amish

#### 2.7.1 ∣ P Variants With Known or Suspected Clinical Relevance

To evaluate the clinical impact of P variants on the health of the Amish and UKB Europeans, we cross-referenced all variants sequenced in the Amish and UKB European participants with the ClinVar database to identify those in ClinVar classified as P or LP and had a support level of at least two stars. A ClinVar designation of two stars indicates submission to ClinVar by multiple submitters that includes a description of the criteria used to classify the variant and agreement of classification among the submitters. We did not include variants with conflicting interpretations in this analysis. In the Amish, we included an additional set of population-specific variants not meeting this ClinVar criteria but designated as P/LP by the Clinic for Special Children (CSC), a clinical and research facility in Lancaster with the mission of diagnosing and treating Amish and Mennonite children with genetic disorders (Morton et al. 2003; Strauss and Puffenberger 2009). To meet the demand for carrier testing in Plain populations (i.e., Amish and Mennonite) throughout North America, the CSC has developed with their partners a next-generation sequencing panel to provide comprehensive coverage for critical P alleles (Crowgey et al. 2019). This panel, named the Plain Insight Panel (PIP) and now in its third version, contains P variants informing the management of newborns, as well as variants that cause dominantly inherited, potentially preventable diseases later in life. PIP variants are classified by CSC as Tier 1, which contain well-documented disease variants that manifest in childhood and/or adulthood, Tier 2, which include genetic variants that lack clinical support but are suspected to cause disease based on predicted deleterious gene effect (i.e., nonsense, frameshift, or canonical splice site variants) or missense variants with P/LP assertions in ClinVar for other populations, or Tier 3, which include variants considered to be genetic modifiers or pharmacogenetic risk variants that alone do not affect health. We considered only CSC variants classified as Tier 1 in our analysis (see Table S2). All CSC Tier 1 variants are based on variant curation and clinical assessments, and all AR Tier 1 variants have at least one documented symptomatic patient followed at the CSC (or one of its sister clinics). Inheritance patterns for the P/LP variant-associated disorders were obtained from Online Mendelian Inheritance in Man (OMIM). Table S3 shows the OMIM inheritance patterns we used for our analysis of autosomal dominant and recessive disorders.

We first compared the proportion of Amish and UKB European subjects heterozygous or homozygous for one or more of the aforementioned variants, using the chi-square test. Because of the endogamous nature of the Amish community, we then calculated the proportion of Amish couples in our sample (*n* = 2141) who were carrier couples for either autosomal dominant or autosomal recessive disorders. We defined a carrier couple as a husband–wife pair in our sample in whom either parent had at least one copy of a P/LP variant associated with a dominant disorder or both parents had at least one copy of the same P/LP variant associated with a recessive disorder.

#### 2.7.2 ∣ P Variants in Clinically Actionable Genes

To evaluate the clinical impact of limited genetic diversity and drift, we compared the number of individuals with P/LP variants in 81 genes deemed to be clinically actionable by the ACMG (Version 3.2) (Miller et al. 2023) between Amish and UKB Europeans. For these analyses, we used the Franklin platform for identifying and classifying variants of clinical importance (Rodrigues et al. 2022). This platform utilizes multiple databases and resources that incorporate assessment of population frequency, predicted variant effect, functional evidence, and disease and phenotype databases to classify variants in accordance with the ACMG/AMP guidelines for variant interpretation. For both Amish and UKB, we extracted all sequenced variants within the 81 ACMG V3.2 actionable gene panel and obtained variant interpretations (annotations obtained from Franklin between February 14, 2025, and February 28, 2025).

#### 2.7.3 ∣ Genetic Association Analysis of Cardiometabolic and Bone Health Traits in 7221 Amish

We have previously conducted genome-wide association studies (GWAS) on a range of cardiometabolic and bone health-related traits in the Amish. To illustrate the clinical relevance of the limited genetic diversity within our cohort of 7221 individuals, we present a summary of impactful associations, providing their effect sizes and contrasting allele frequencies of these variants between the Amish and non-Finnish Europeans as documented in the Genome Aggregation Database (gnomAD) (Chen et al. 2024).

## 3 ∣ Results

### 3.1 ∣ Demographics and Relatedness

**FIGURE 1 ∣:**
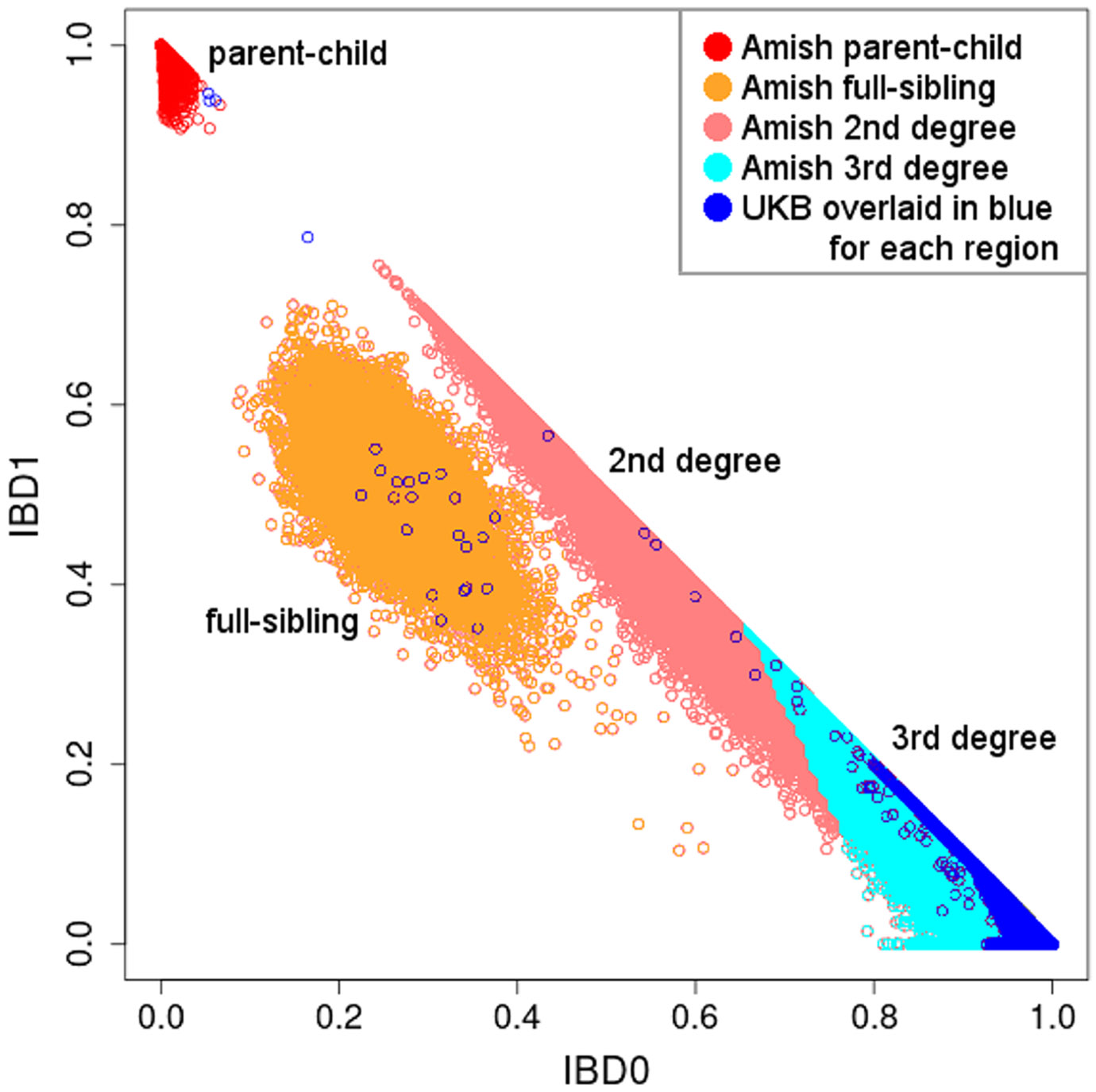
Pairwise relatedness among 7221 Amish and 7221 UKB individuals. IBD, identity by descent.

The Amish WES cohort ranged in age at recruitment from 18 to 103 years compared to ages 40–69 years for the UKB European cohort. As indicated in Figure 1, Amish individuals are more related compared to UKB Europeans. The mean proportion of alleles that are identical by descent (IBD) among Amish relative pairs is 0.077, which is equivalent to a pairwise relationship slightly closer than second cousins (i.e., individuals who share one great-grandparent), who share 0.0625 of their alleles in common. This estimate decreased only slightly after excluding first-degree relative pairs (mean IBD = 0.073). In comparison, the mean IBD among UKB Europeans without the exclusion of first-degree relatives was 0.014. The high relatedness among the Amish is also highlighted by the fact that 78% of these 7221 individuals share one of only eight family names (after combining variations in family name spelling), consistent with the relatively small number of Amish who founded the Lancaster settlement and subsequent genetic drift leading to over-representation of some names and under-representation of others.

### 3.2 ∣ Characterization of Exome Variants

#### 3.2.1 ∣ Numbers and Types of Exonic Variants

**FIGURE 2 ∣:**
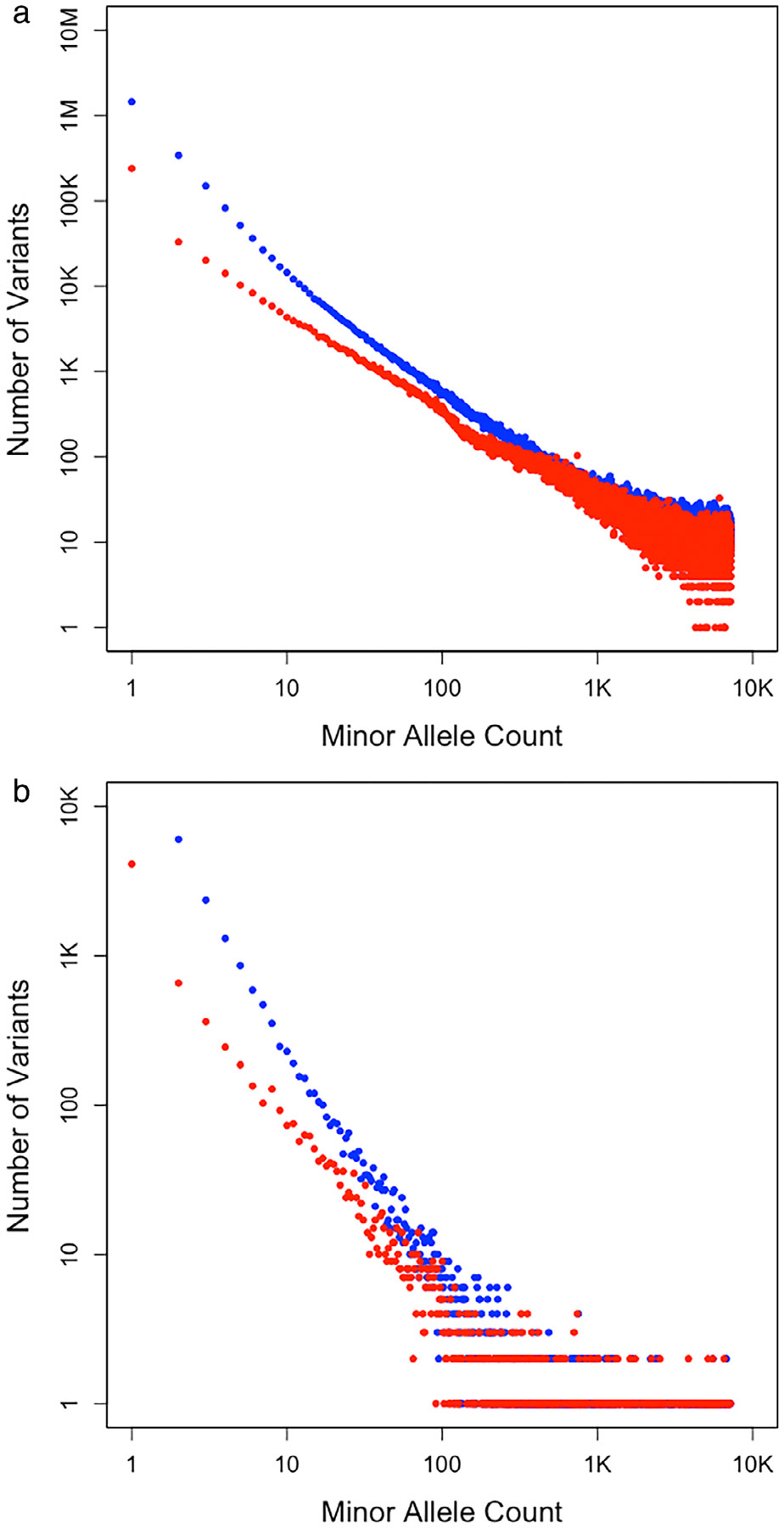
Comparison of minor allele counts for ES variants between 7221 Amish (red) and 7221 UKB (blue) for all variants (a) and pLOF variants (b).

We compared the numbers and types of variants between Amish and UKB Europeans matched for sample size. The Amish had 146,334 exonic/splicing variants compared to 1,067,781 in UKB Europeans (Table 1). The larger number of variants seen in the UKB European subset (7.3 times as many) was observed mainly for variants with allele frequencies < 1%, for whom UKB Europeans had 10.9 times as many variants (Table 1); the disproportionate number of variants in the UKB European subset was especially apparent for ultra-low frequency variants (Figure S1a). By contrast, the number of variants with allele frequencies > 1% was approximately similar between the two groups (53,460 in Amish vs. 57,012 in UKB Europeans) (Table 1 and Figure 2a). The vast majority of exonic and splicing variants present in the Amish were single-nucleotide substitutions (93% single-nucleotide substitutions, 5% indels, 2% multiallelic), and these proportions differed little from those observed in UKB Europeans (94%, 4%, and 2%, respectively) (Table 1).

#### 3.2.2 ∣ pLOF Variants

The UKB European subset had 6.0 times as many pLOF variants as Amish (49,813 vs. 8297) (Figure 2b), with the excess again occurring mostly for variants with MAF < 1% (Table 1 and Figures S1b and S2a). pLOF variants occurred in 5429 unique genes in the Amish and in 13,841 unique genes in UKB Europeans. Among pLOF variants in the Amish, 53% were frameshift, 25% stop gain, 11% splice acceptor, 8% splice donor, and 2% stop loss. This distribution differed significantly from that observed in UKB Europeans (49%, 35%, 7%, 9%, and 1% for frameshift, stop-gain, splice acceptor, splice donor, and stop-loss variants, respectively; *p* < 0.001; Table 1 and Figure S2b). On average, each Amish individual was homozygous for 18.9 pLOF variants, and each UKB European individual was homozygous for 19.9 pLOF variants (*p* < 0.001).

Following successive downsampling of the UKB European sample, we observed that a sample of only 507 UKB Europeans yielded a total of 8299 unique pLOF variants, suggesting that the 7221 Amish exomes in our sample may have been derived from ~500 independent founders. This estimate of ~500 is roughly consistent with the previously pedigree-based analysis of Lee et al. (2010), suggesting that the Lancaster settlement was founded by ~400 settlers.

#### 3.2.3 ∣ Allele Frequencies in Amish and UKB Europeans

As shown in Figure S3a (all variants) and Figure S3b (pLOF variants only), the frequencies of common alleles (MAF > 0.10) were highly correlated between Amish and UKB Europeans, although there is considerable variability in MAF between the two populations. At lower MAF, there is greater divergence in allele frequencies between the two groups (Figure S3c,d) as many variants present in UKB Europeans are lost in the Amish, consistent with the past bottleneck event, and some variants present in both groups occur at markedly higher or lower frequency in the Amish, consistent with genetic drift.

Over 75% of the pLOF variants present in the Amish were not present in UKB Europeans (6516/8297, or 78.5%), although the vast majority of these (6365 variants, or 96.7%) had MAF < 0.01. Notably, there were 103 pLOF variants with a frequency > 0.02 in the Amish, all of which were absent in UKB Europeans or had a frequency < 0.001.

### 3.3 ∣ Clinical Impact of P/LP Variants

#### 3.3.1 ∣ P/LP Variants With Suspected Clinical Relevance in the Amish and UKB Europeans

**FIGURE 3 ∣:**
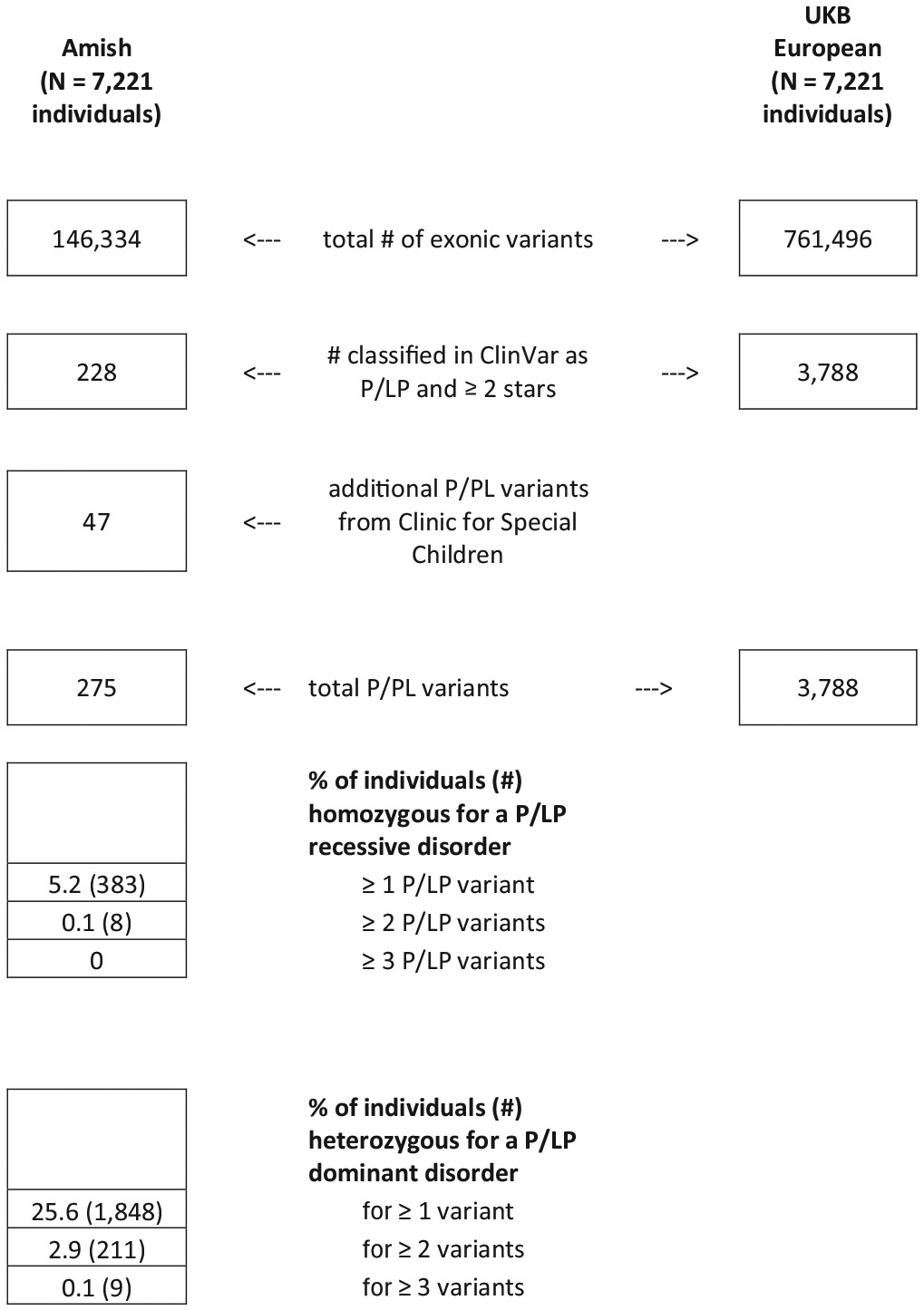
Identification of pathogenic (P) and likely pathogenic (LP) variants in Amish and UKB Europeans and proportions of Amish homozygous for recessive disorder-associated P/PL variants and heterozygous for dominant disorder-associated P/PL variants.

We identified 230 variants in the Amish and 3788 variants in UKB Europeans that had ClinVar classifications of P or LP and a support level of two stars or higher (Figure 3). We removed two of these because they were annotated in ClinVar as a low penetrance risk factor and/or drug response variant (*F5 c.1601G>A, p.Arg534Gln (also known as Factor V Leiden*) and *F2* c.*97G>A), both of which increase the risk of venous thromboembolic disease. However, it is notable that the Amish are enriched for F5 p.Arg534Gln (MAF = 0.103 compared to 0.022 in UKB Europeans). We identified an additional 106 Tier 1 (P/LP) variants present in our Amish cohort from the CSC PIP panel. Of these, 59 (56%) were classified as P/LP by ClinVar and 47 (44%) were not. We included these additional 47 for analysis, bringing the total of Amish ClinVar/PIP variants to 275. These variants have been implicated in a wide variety of clinical disorders, many of which are involved in metabolic function and affect a variety of organ systems (see Table S2). As expected, concordance of the P/LP classifications between ClinVar and the Franklin platform was higher for the ClinVar than for the PIP-annotated variants (Figure S4).

The 275 P/LP variants in the Amish fell within 228 different genes, whereas the 3788 P/LP variants in UKB Europeans fell within 1141 different genes. Among the Amish, 61.1% (168 out of 275) of the P/LP variants were unique to the Amish, and these fell in 62 genes for which there were no P/LP variants observed among UKB Europeans. Twenty-seven of the P/LP variants observed in Amish had a minor allele frequency > 2.0%; of these 27 variants, 22 were either absent among UKB Europeans or had MAF < 0.1%. Most, but not all, of these variants are highly enriched in the Amish compared to the Genome Aggregation Database (gnomad), with 145 of the 275 variants (52.7%) having a 10-fold or higher enrichment and 190 (69.1%) variants having a 3-fold or higher enrichment.

#### 3.3.2 ∣ Clinical Impact of P/LP Variants in the Amish

Approximately 97% of all Amish and UKB Europeans were heterozygous for at least one P/LP variant (Table S4). Amish individuals carried on average 3–4 P/LP variants and were more likely to be heterozygous for multiple P/LP variants compared to UKB Europeans (e.g., 30.0% of Amish vs. 25.0% of UKB Europeans were heterozygous for 5 or more P/LP variants; *p* < 0.001). In total, 5.2% of Amish individuals were homozygous for a P/LP variant in a recessively acting autosomal disease gene (Figure 3). An additional 1.1% of individuals (*n* = 84) carried two different P/LP variants in a recessively acting autosomal disease gene, but we did not determine whether the two variants are in different copies of the gene (i.e., haplotype phasing) in a manner consistent with recessive inheritance. The most common recessive P/LP variants were *SERPINA1* (p.E288V), associated with alpha-1-antitrypsin deficiency, *EXOSC3* (p.V80F), associated with pontocerebellar hypoplasia type 1B, and *TJP2* (p.V52A), associated with familial hypercholanemia. Eight individuals were homozygous for two different recessive P/LP variants.

As we did not perform detailed medical assessments on P/LP variant carriers, we sought evidence for selection against the inclusion of individuals homozygous for a P/LP variant in a recessively acting gene in our sample. Across all autosomal recessive genes, we observed a deficit of homozygous variant genotypes beyond what would be expected by chance (383 homozygous genotypes observed across 197 recessively acting genes compared to 492 expected under Hardy–Weinberg equilibrium [HWE]), consistent with a significant health impairment and ascertainment bias against participation in our community-based studies (HWE *p* value < 0.0001). The major drivers of this imbalance were *EVC* c.1886+5G>T, *LONP1* p.Arg721Gly, and *DHODH* p.Arg135Cys, all having minor allele frequencies > 0.04 (Table S5).

In addition, 25.6% of Amish individuals were heterozygous for at least one dominant P/LP variant (Figure 3). The most common of these were *APOB* (p.Arg3527Gln, MAF = 6.7%), associated with familial hypercholesterolemia, *THAP1* (p.Phe45Leufs*30, MAF = 1.3%), associated with primary torsion dystonia, and *KCNQ1* (p.Thr224Met, MAF = 1.2%), associated with long QT syndrome (LQTS). A full list of P variants present in the Amish, including numbers of heterozygotes and homozygotes for each, is provided in Table S2.

#### 3.3.3 ∣ Frequency of Carrier Couples

The high degree of relatedness within the Amish community is also observed among the spouse pairs (*n* = 2141), for whom 71% have a coefficient of relatedness ≥ 0.0625. The mean proportion of alleles identical by descent among spouse pairs was 0.073 (median: 0.071), indicating that Amish couples in this community are on average closer than second-degree cousins. These patterns are observed despite Amish customs that generally avoid mate selection between close relatives.

Both spouses were heterozygous for the same P/LP recessive variant in 24.3% (520 out of 2141) of couples, meaning that each of their children was at ~25% risk of inheriting two copies of that variant. In 43.6% of couples (934 out of 2141), at least one spouse was heterozygous for a P/LP dominant variant, meaning that each of their children was at ~50% risk of inheriting a dominant P/LP variant. Moreover, 9.6% of couples were at risk of transmitting P/LP alleles for at least two different dominant disorders to their children, and 2.4% of couples were at risk of transmitting P/LP alleles for at least two different recessive disorders to their children (Table 2).

#### 3.3.4 ∣ Variants in Clinically Actionable Genes

Among the 81 genes in the ACMG V3.2 panel deemed clinically actionable, Franklin classified 26 (out of 5092 total variants in these genes) as P, including the *KCNQ1* p.Thr224Met variant, which we previously reported to be strongly associated with longer QT interval and LQTS in the Amish with functional evidence of a near complete loss of protein function (Streeten et al. 2020). These 26 variants fell in 20 different genes. Among UKB Europeans, Franklin classified 261 (out of 22,713 total variants in these genes) as P, with these falling in 45 different genes.

Our analyses detected one variant (*HFE* 187C>G; p.His63Asp) that was highly common in both Amish (MAF = 6.0%) and UKB Europeans (MAF = 15.0%). Variants in *HFE* have been associated with hemochromatosis. Because of its high frequency in both populations, we evaluated its clinical significance in a recently published analysis from the UKB. Unlike another reportable variant in this gene described in this paper, homozygosity for the *HFE* p.H63D variant was not associated with any clinical phenotypes (mortality, fatigue, depression, liver disease or cirrhosis, or joint replacements) in this analysis (Lucas et al. 2024). This variant was classified as P by Franklin, although it is classified as “Pathogenic/Likely pathogenic/Pathogenic, low penetrance; other” in ClinVar. Based on this information, we have provided counts of the number of reportable variant carriers, both including and excluding the *HFE* p.His63Asp variant.

Overall, 17.4% of Amish had at least one reportable P variant compared to 8.3% of UKB Europeans. In the Amish, more than five heterozygotes were observed for nine different variants: *APOB* p.Arg3527Gln, *KCNQ1* p.Thr224Met, *APC* p.Ser837*, *APC* c.423–3T>A, *MUTYH* p.Ala357Profs*23, *COL3A1* c.1609–2A>C, *LMNA* p.Arg190Trp, *MSH2* c.942+2del, and *APOB* p.Lys3067Argfs*2. Among Amish, 15.0% had either the FH-causing *APOB* p.Arg3527Gln variant (carried by 13.1% of Amish) or the LQTS-causing *KCNQ1* p.Thr224Met variant (carried by 2.5% of Amish). Among UKB Europeans, the most common reportable variants were the *HNF1A* p.Pro-289Alafs*28, *MUTYH* p.Tyr151Cy, *ATP7B* p.Gly869Arg, and *MSH6* p.Phe1088Leufs*5 variants, occurring in 48, 26, 22, and 22 individuals, respectively. A complete list of all reportable variants is provided in Table S6 (Amish) and Table S7 (UKB). Including the *HFE* p.His63Asp variant, 28.9% of Amish had at least one reportable P variant compared to 36.2% of UKB Europeans.

#### 3.3.5 ∣ Genetic Association Analysis of Cardiometabolic and Bone Health Traits in 7221 Amish

We identified numerous rare and low-frequency variants significantly enriched in the Amish compared to the gnomAD reference population that were associated with a range of cardiometabolic and bone health-related traits. Table 3 summarizes key associations, including effect sizes, allele frequencies, and *p* values.

Notable findings include:

- *APOB* rs5742904 and *APOOP1* rs898956003: Each copy of the risk allele was associated with 62% and 11% higher levels of LDL cholesterol, respectively.
- *APOC3* and *B4GALT1* rare variants: These were cardioprotective, associated with a 48% lower triglyceride response to an oral fat tolerance test and a 10% reduction in LDL cholesterol levels, respectively.
- *KCNQ1* rs199472706: Associated with a 5% longer QT interval, suggesting potential implications for cardiac repolarization.
- Variants in other genes:
  - *ABCG8*: Linked to 35% higher levels of sitosterol.
  - *LIPE*: Associated with increased risk of dyslipidemia, hepatic steatosis, and diabetes.
  - *COL1A2*: Associated with osteogenesis imperfecta and increased risk of bone fracture.
  - *SLC12A3*: Associated with 4% lower serum potassium.

## 4 ∣ Discussion

The genetic architecture of the OOA is profoundly influenced by its unique population history, shaped principally by the small number of founders who immigrated to Lancaster County 12–14 generations ago to establish the Lancaster settlement. This migration and subsequent endogamy constituted a bottleneck event leading to a profound reduction in genetic diversity, as evidenced by the Amish having only 19% as many exonic and splicing variants as UKB Europeans. This bottleneck, combined with the effects of genetic drift over subsequent generations, has substantially influenced their genetic makeup. Consistent with the Amish being of European ancestry, pLOF variants common in Amish tended also to be present in UKB, and vice versa. Notably, many of the variants that are present in the Amish, particularly some low-frequency variants in the general population, have drifted to higher frequencies, including many that have, or may have, clinical impact.

Our study shows that ~500 UKB individuals possess a similar number of exonic and splicing variants as our full sample of 7221 Lancaster County Amish. This difference is primarily due to the fact that the Amish community has only hundreds of immigrant founders, with 128 founders accounting for over 95% of the mean relative founder contribution to living individuals (Lee et al. 2010). Also contributing to this difference is that the ancestors of the Amish founding immigrants who died in Europe ~15 generations ago were all located in present-day Switzerland, Germany, and France, while the 15-generation ancestors of UKB participants likely were more spread out geographically and hence genetically.

The clinical impact of these demographic forces on the Amish community is consequential. Overall, 5.2% of Amish individuals were homozygous for a recessive P/LP variant, and 25.6% of individuals were heterozygous for at least one dominant P/LP variant. We have previously shown the *APOB* p.Arg3527Gln variant to be associated with a 76 mg/dL increase in LDL cholesterol per allele in the Amish and a 9.3-fold higher odds of having severe coronary artery calcification (Shen et al. 2010). Its frequency in the Amish is enriched 200-fold compared to UKB Europeans (MAF in UKB = 0.03%). A second commonly occurring variant in the Amish (MAF = 1.2%), *KCNQ1* p.Thr224Met, is not present in the 7221 UKB Europeans. Due to these variants, familial hypercholesterolemia was present in 13.1% of Amish individuals, and 2.5% of individuals were at risk for LQTS and sudden cardiac death.

The high frequency of P/LP variants presents a particularly high burden to Amish families. In 24% of Amish couples, both spouses were heterozygous for the same recessive P/LP variant, and in 44% of couples, at least one spouse was heterozygous for a dominant P/LP variant. This high genetic load has sparked interest among some in the Plain communities for carrier screening of known recessive diseases in couples contemplating having children. At least two main benefits are ascribed to carrier screening. The first is that for some autosomal recessive disorders, early detection can prompt initiation of therapy in the newborn, which can have profound effects on health. One example is glutaric acidemia (glutaryl-CoA dehydrogenase [GCDH] deficiency), a neurometabolic disorder caused by a deficiency or absence of the mitochondrial enzyme GCDH that is involved in lysine metabolism, but whose neurologic sequela can be avoided by treatment with a lysine-free metabolic diet if initiated in the newborn period (Boy et al. 2023). A second example, occurring in the Lancaster Mennonite but not Amish, community, is maple sugar urine disease, a neurological disorder stemming from a deficiency of an enzyme complex (branched-chain alpha-keto acid dehydrogenase) that is required to break down three branched-chain amino acids. The diagnosis and initiation of dietary therapy in newborns within the first day or two of life can lead to prevention of acute intoxication in the newborn period through correction of imbalances in plasma amino acids and thereby prevent or reverse life-threatening cerebral edema (Morton et al. 2002). A second benefit of carrier screening in this community is that a perinatal diagnosis of a life-threatening genetic disease, even if no treatment were available, can be empowering for some parents in allowing them to prepare for their infant's death while avoiding extensive medical testing and expense that would not affect their infant's health outcome. For these couples, carrier screening can provide a choice between having their baby spend their short life in the hospital or at home with the parents and family. Finally, as the Amish typically come from large sibships and have on average 5–7 children, carrier status identified in one Amish adult often leads to cascade testing of other adult relatives and further identification of couples at risk for children with disorders known to segregate within the community.

Through our prior genetic association analyses, we have identified multiple Amish-enriched variants associated with a range of cardiometabolic and bone-health-related traits (see Table 3). Several of these variants have direct translational relevance for risk reduction. For instance, population and cascade screening for hypercholesterolemia-associated variants could help identify individuals who may benefit from cholesterol-lowering therapies. Additionally, dietary restriction of plant sterols may reduce cardiovascular risk in individuals homozygous for the *ABCG8* variant associated with sitosterolemia. For carriers of the *KCNQ1* rs199472706 variant linked to LQTS, risk mitigation strategies—such as lifestyle modifications and pharmacologic interventions—may help prevent sudden cardiac death. The strong community support among the Amish aids genetically vulnerable community members to adhere to special diets or other lifestyle interventions that counterbalance the risk.

Despite the high burden of disease conferred by their genetics, there are other factors that substantially favorably impact the health of the Amish. For example, Amish females rarely smoke, and among male smokers, the intensity of smoking is modest (Reed et al. 2017). We have previously shown that the Lancaster Amish have a relatively low prevalence of type 2 diabetes, hypertension, and high cholesterol (despite a high frequency of familial hypercholesterolemia) compared to non-Amish Europeans in the United States (He et al. 2020) as well as a lower prevalence of hip fracture (Streeten et al. 2004). Moreover, mortality rates in the Amish are approximately similar to those observed in European ancestry participants of the Framingham Heart Study, although this similarity was conditional on having lived until age 50 years and so does not include early deaths (Mitchell et al. 2012). We have speculated that the active Amish lifestyle and perhaps other aspects of Amish life (e.g., social connectedness) have beneficial effects on Amish health.

There are also highly enriched variants in the Amish that are likely beneficial to health. For example, we have previously shown that the *APOC3* p.R19X variant (MAF = 2.3% in Amish vs. < 0.01% in non-Amish Europeans) is associated with a 48% lower triglyceride response to an oral fat tolerance test, a cardioprotective lipid profile, and presumed protection from cardiovascular disease as indicated by lower levels of coronary calcification (Pollin et al. 2008; Reyes-Soffer et al. 2019). Subsequently, studies have shown that *APOC3* LOF variants in aggregate reduce the risk of myocardial infarction by 40% in the general population (Crawford et al. 2014; Jørgensen et al. 2014; TG HDL Working Group of the Exome Sequencing Project, National Heart Lung Blood Institute et al. 2014). We recently showed another Amish-enriched variant, *B4GALT1* p.Asn352Ser (MAF = 5.9% in Amish vs. < 0.000001% in non-Amish Europeans) to be associated with 10% lower LDL cholesterol levels and lower cardiovascular risk, although its impact overall on health is as yet unclear (Montasser et al. 2021).

Because we did not perform comprehensive medical assessments of our Amish study participants, we were unable to directly determine the extent to which P/LP variants manifested with clinical phenotypes. For example, many of the P/LP variants identified through ClinVar have been previously linked to neurodevelopmental disorders, which were not assessed in our studies. It is also possible that individuals affected by such conditions were underrepresented in our sample, as recruitment was based on voluntary participation from the community. It is also possible that some of these P/LP variants exhibited variable penetrance, further complicating phenotype–genetic correlations.

Despite these limitations, we observed an under-representation of homozygous individuals for P/LP variants across all recessively acting genes, consistent with a selection against participation among affected individuals. Furthermore, several of the identified variants are well documented as having low penetrance in both homozygous and heterozygous states (e.g., *HFE* p.His63Asp and *HFE* p.Cys282Tyr, *EXOSC3* p.Val80Phe, *TJP2* p.Val48Ala, *TNFRSF1A* p.Arg121Gln, *MYBPC3* c.3330+2 T>G, and *GBA* p.Asn409Ser). It is also possible that some of these variants were misclassified as P/LP, highlighting the need for ongoing refinement of variant interpretation frameworks.

While this study represents by far the most comprehensive evaluation of genetic variation in a single Anabaptist group, there are limitations to our analysis. Much of our analysis of the clinical impact of P variants is based on the current curation of the ClinVar database, for which only a small proportion of entered variants have undergone careful evaluation and classification by an Expert Panel. As ClinVar is a dynamic database, the criteria that we used to define P/LP (i.e., those classified as P or LP with a level of support of at least 2 stars) will invariably change for some variants as new information becomes available.

A related limitation of our study pertains to the biases inherent in the ClinVar database. ClinVar submissions are heavily weighted towards inclusion of variants in well-studied genes and clinical conditions, variants that have undergone testing in clinical laboratories, and in non-isolated populations. We have attempted to ameliorate some of these biases by including a carefully curated Amish-specific panel of P variants in our analysis that are very rare in European (non-Amish) populations.

Thirdly, as our analysis is based on ES, it was not designed to identify clinically important non-exonic variants (e.g., *RMRP* n.72A>G, causal in the Amish for cartilage-hair hypoplasia; Ridanpää et al. 2003; Rider et al. 2009) or structural rearrangements or large deletions (e.g., in *SMN1* and *SLC12A3*, causal in the Amish for spinal muscular atrophy and Gitelman syndrome, respectively; Carson et al. 2018; Wan et al. 2021).

In a broader context, there are over 600 Amish and other Anabaptist settlements across the United States and Canada (Young Center for Anabaptist and Pietist Studies 2025), each having their own distinct demographic histories and distinct sets of founders. The specific configuration of each settlement's founder event, including the number and relatedness of the founders and the duration of the bottleneck, varies, leading to differing degrees of reduced genetic diversity among groups. Furthermore, random genetic drift operates independently in each settlement, contributing to the settlement-specific enrichment of variants over generations. As a consequence, many of the enriched alleles that are present in one settlement are likely to be different between settlements. Thus, gene discovery efforts in each of these unique communities may uncover additional genes with P variants with important implications for population genomic health in these communities.

In summary, we report a comprehensive catalog and frequencies of Mendelian and clinically actionable variants in the OOA Lancaster, PA settlement. In addition to providing opportunities for novel gene discoveries that inform health and disease, once known, founder populations such as the Amish provide an ideal setting in which to implement population genomic medicine. Lessons learned from such implementation will have far-reaching implications for the practice of genomic medicine in more diverse and general populations.

## Supplementary Material

Suppl Figures

Suppl Tables

Additional supporting information can be found online in the Supporting Information section. **Figure S1:** Number of variants in Amish (red) and UKB (blue) with minor allele counts ranging from 1 to 10 for all variants (a) and pLoF variants only (b). **Figure S2:** Distribution (a) and types (b) of pLOF variants in the Amish and UKB. **Figure S3:** Comparison of allele frequencies between 7221 Amish and 7221 UKB for all WES variants (a) and pLOF variants (b), and of allele frequencies with MAF < 0.10 for all WES variants (c) and pLOF variants (d). **Figure S4:** Concordance of P/LP classifications with the Franklin platform between 228 P/LP variants from ClinVar and 109 P/LP variants from PIP. **Table S1:** Contributing studies to the Amish Research Program that include participants with WES. **Table S2:** 275 Pathogenic Variants Identified in the Amish through ClinVar (*n* = 230) and Clinic for Special Children (*n* = 45). **Table S3:** Correspondence of OMIM inheritance patterns to the autosomal recessive and autosomal dominant inheritance patterns used for analysis in this study. **Table S4:** Proportion of Amish and UKB subjects (*n* = 7221 each) heterozygous or homozygous for a clinically important pathogenic variant. **Table S5:** Numbers of observed and expected homozygotes for P/LP variants in recessively acting genes in 7221 Amish. **Table S6:** Pathogenic variants in 81 clinically actionable genes from ACMG 3.2 in 7221 Amish. **Table S7:** Pathogenic variants in 81 clinically actionable genes from ACMG 3.2 in 7221 UKB Europeans.

## Figures and Tables

**TABLE 1 ∣:** Number of variants (total and in exonic/slicing regions) observed in 7221 Amish and 7221 UKB Europeans.

	Amish		UKB	
	Autosomal variants in WES		Autosomal variants in WES	
	ALL	MAF < 1%	ALL	MAF < 1%
Total variants	586,692	449,307	2,581,693	2,397,631
Exonic and splicing regions	146,334	92,874	1,067,781	1,010,769
Variant type^a^				
SNV	138,224	85,705	1,023,980	968,512
Indels	8110	7169	43,801	42,257
Multi-allelic	2549	2397	20,104	18,934
Predicted function				
Synonymous	53,588	27,713	349,160	321,496
Missense	79,140	53,680	638,101	611,612
LOF (any transcript)	8297	7600	49,813^b^	48,909^b^
Frameshift	4429	4096	24,040	23,540
Stop gained	2081	1826	17,232	16,989
Splice acceptor	927	890	3549	3483
Splice donor	694	646	4335	4270
Stop lost	166	142	660	630

a Sum of SNVs + Indels = exonic and splicing region variants.

b The total number of LOF variants in the UKB does not equal the sum of each type because three variants—1 Frameshift, 1 Stop gain, and 1 Stop loss—were also classified as Splice Donor variants.

**TABLE 2 ∣:** Number (and percent) of Amish couples at risk for transmitting a genetic disorder to their children.^a^

At risk of transmitting P/LP variant(s) at	Transmission of a dominant disorder	Transmission of a recessive disorder
	No. of carrier couples (%)	No. of carrier couples (%)
1 Gene	43.6% (934)	24.3% (520)
2 Genes	9.5% (204)	2.3% (49)
3 Genes	0.9% (20)	0.1% (3)
4 Genes	< 0.1% (1)	< 0.1% (1)

a Carrier couples defined as couples in whom at least one spouse carries one or more copies of a P/LP variant for a dominant disorder, or both spouses carry the same P/LP variant for a recessive disorder.

**TABLE 3 ∣:** Variants enriched in the Amish that are associated with cardiometabolic and bone health traits.

Gene	Variant	Variant type	MAF in Amish	MAF in non-Finnish EUR (from gnomAD)	Enrichment (Amish MAF/ EUR MAF)	Phenotype and effect size	Citation
*APOB*	rs5742904	SNV	0.067	0.000479	140	76 mg/dL higher (62%) LDL cholesterol levels	Shen et al. (2010)
*APOC3*	rs7635203	SNV	0.023	0.000544	42	48% lower triglyceride response to OFTT	Pollin et al. (2008)
*ABCG8*	rs137852988	SNV	0.018	0.00009	200	0.13 mg/dL (35%) higher sitosterol levels	Horenstein et al. (2013)
*LIPE*	rs587777699 (p.V767Gfs*102)	Frameshift deletion	0.018	0.00044	41	Dyslipidemia, hepatic steatosis, systemic insulin resistance, and diabetes	Albert et al. (2014)
*B4GALT1*	rs551564683 (p.Asn352Ser)	SNV	0.059	0.000015	3934	13 mg/dL (10%) lower LDL cholesterol and 29 mg/dL (10%) lower plasma fibrinogen	Montasser et al. (2021)
*APOOP1*	rs898956003	SNV	0.075	0.000044	1638	15 mg/dL (11%) higher LDL cholesterol levels	Montasser et al. (2018)
*KCNQ1*	rs199472706	SNV	0.011	NF	∞	20.2 ms longer (5%) QT interval	Streeten et al. (2018)
*SLC12A3*	rs200697179 (p.R642G)	SNV	0.014	0.000044	313	0.15 mmol/L (4%) lower serum potassium	Wan et al. (2021)
*C0L1A2*	rs72658160 (p.Gly349Cys)	SNV	0.0008	NF	∞	0.344 g/cm^2^ (46%) lower hip BMD; Osteogenesis imperfecta	Daley et al. (2009)

Abbreviations: BMD = bone mineral density; NF = not found; OFTT = oral fat tolerance test; SNV = single-nucleotide polymorphism.

## Data Availability

The data that support the findings of this study are available on request from the corresponding author. The data are not publicly available due to privacy or ethical restrictions. A full list of all P/LP variants identified in the Amish is provided in Table S2.
