# Supplementary material for: Exonic Variation and Its Clinical Impact in 7221 Old Order Amish
Source: Am J Med Genet A. Author manuscript; Available in PMC 2026 Jul 21. (PMC13386165; doi:10.1002/ajmg.a.64212)

Exonic Variation and its Clinical Impact in 7,221 Old Order Amish

Mitchell et al.

Supplemental Figures:

Figure S1. Number of variants in Amish (red) and UKB (blue) with minor allele counts ranging from 1 to 10 for all variants (Fig S1a) and pLoF variants only (Fig 1Sb)

Figure S2. Distribution (Fig S2a) and types (Fig 2b) of pLOF variants in the Amish and UKB

Figure S3. Comparison of allele frequencies between 7,221 Amish and 7,221 UKB for all WES variants (Fig 3a) and pLOF variants (Fig 3b) and of allele frequencies with MAF < 0.10 for all WES variants (Fig 3c) and pLOF variants (Fig 3d)

Figure S4. Concordance of P/LP classifications with Franklin platform between 228 P/LP variants from ClinVar and 109 P/LP variants from PIP

Figure S1. Number of variants in Amish (red) and UKB (blue) with minor allele counts ranging from 1 to 10 for all variants (Fig S1a) and pLoF variants only (Fig 1Sb)

Fig S1a

Fig S1b


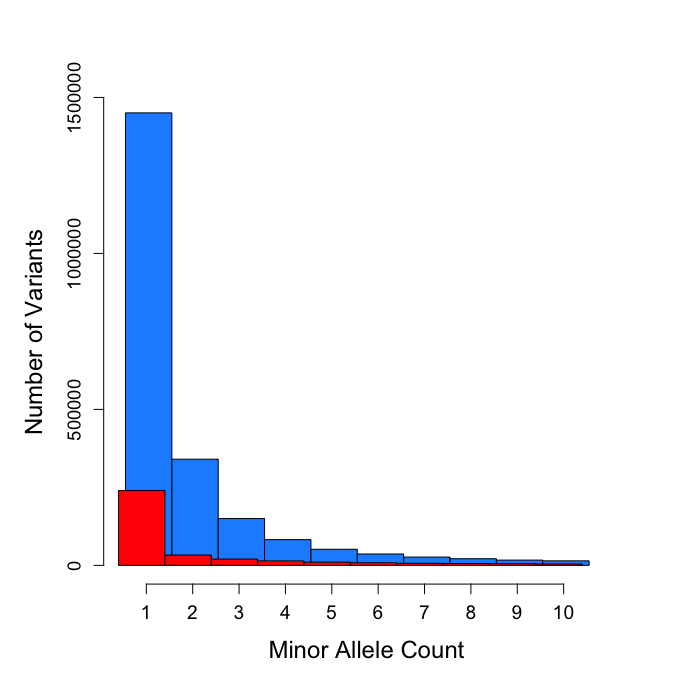

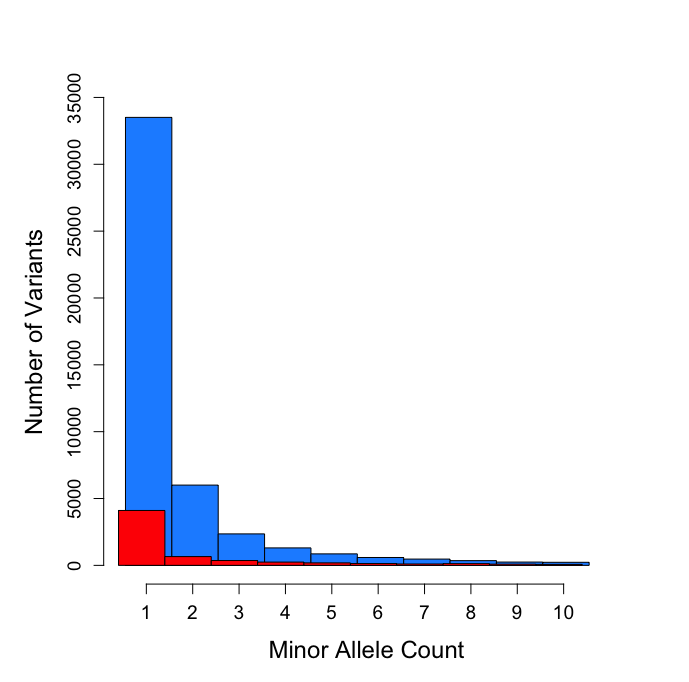


Figure S2. Distribution (Fig S2a) and types (Fig 2b) of pLOF variants in the Amish and UKB

Fig S2a

Fig S2b

Figure S3: Comparison of allele frequencies between 7,221 Amish and 7,221 UKB for all WES variants (Fig 3a) and pLOF variants (Fig 3b) and of allele frequencies with MAF < 0.10 for all WES variants (Fig 3c) and pLOF variants (Fig 3d)

Fig S3b

Fig S3a


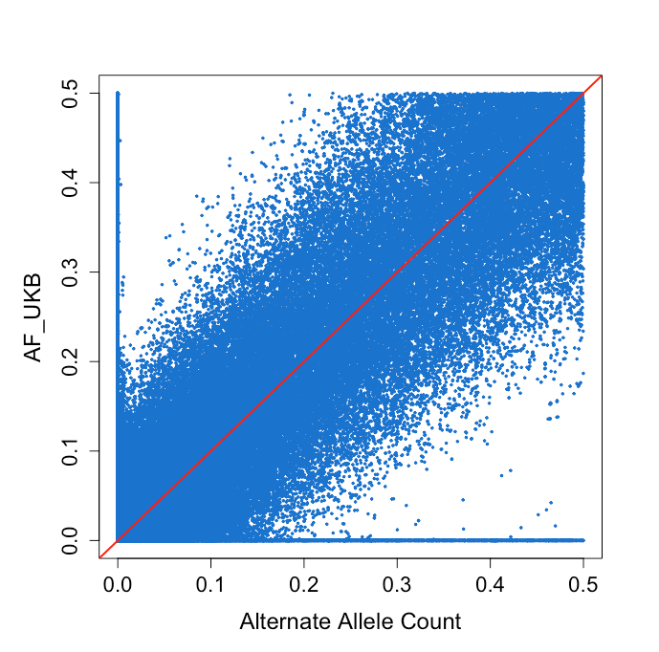

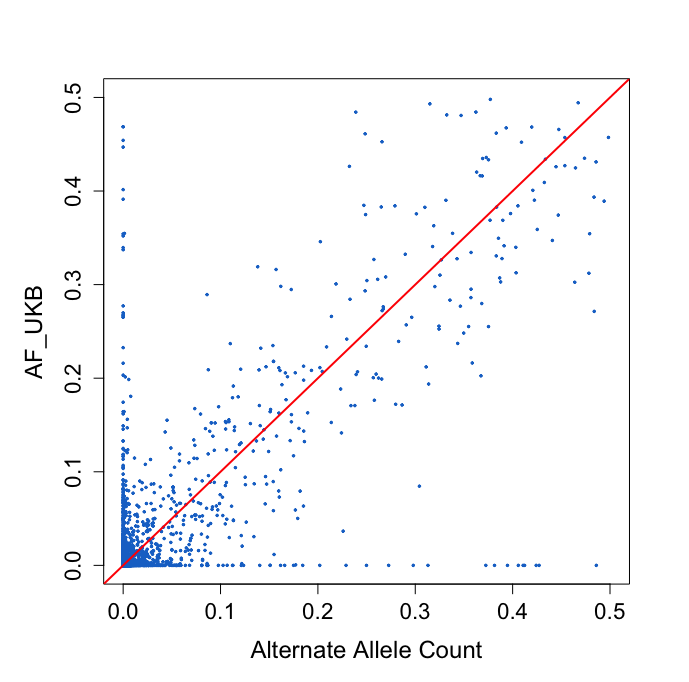


Fig S3c Fig S3d


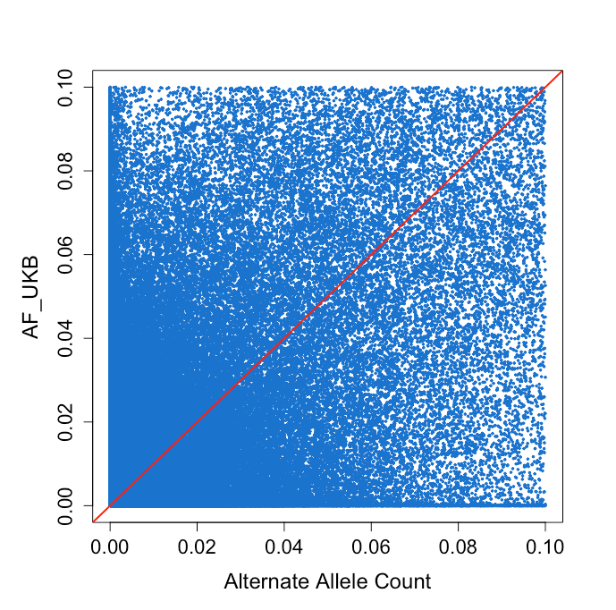

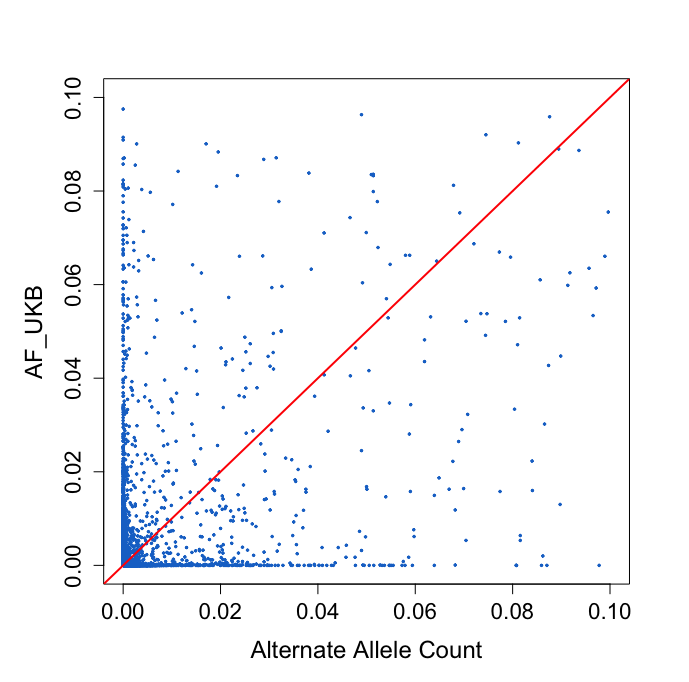


Figure S4. Concordance of P/LP classifications with Franklin platform between 228 P/LP variants from ClinVar and 109 P/LP variants from PIP


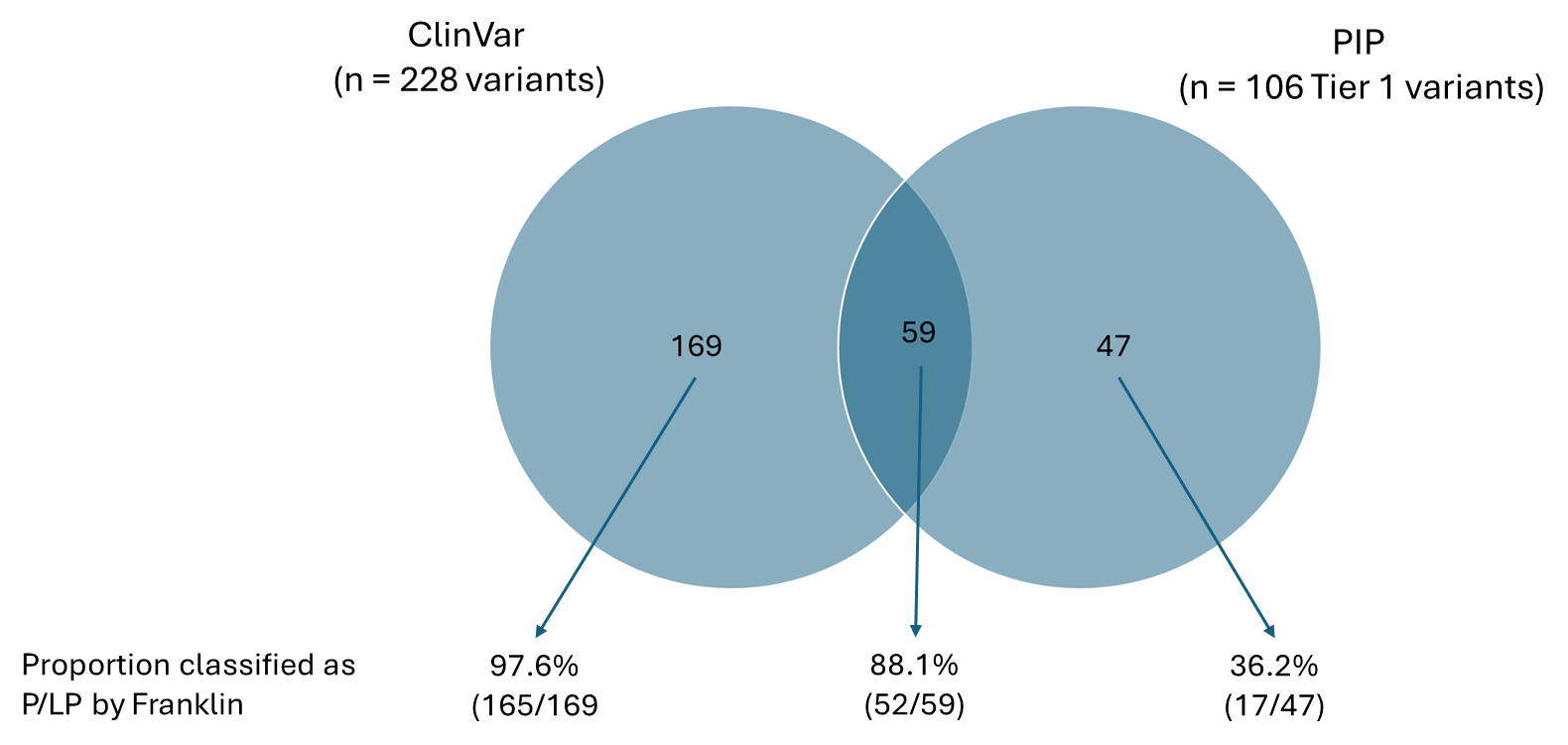

Supplement: Suppl Figures [file NIHMS2192154-supplement-Suppl_Figures.docx]
